# Mining and analysis of drug-induced shock adverse reactions: A comprehensive pharmacovigilance study based on the US FAERS database

**DOI:** 10.1371/journal.pone.0334785

**Published:** 2025-11-13

**Authors:** Junhua Lai, YiPing Pan, JunTao Hu, ZhanHong Tang

**Affiliations:** Intensive Care Unit, The First Affiliated Hospital of Guangxi Medical University, NanNing, GuangXi, China; University of Science and Technology of Fujairah, YEMEN

## Abstract

**Background:**

Shock is a life-threatening clinical condition characterized by high morbidity and mortality. Drug-induced shock represents a complex subset of adverse drug reactions that has not been systematically investigated on a large scale. Comprehensive pharmacovigilance analyses are needed to identify high-risk drugs and drug combinations.

**Method:**

We conducted a retrospective pharmacovigilance analysis using the FDA Adverse Event Reporting System (FAERS) covering the period from 2004Q1 to 2024Q2. Shock-related events were extracted using standardized MedDRA preferred terms. Data deduplication followed FDA guidelines, and four complementary signal detection methods—reporting odds ratio (ROR), proportional reporting ratio (PRR), information component (IC), and empirical Bayes geometric mean (EBGM)—were applied. Drug classification was performed using the Anatomical Therapeutic Chemical (ATC) system, and drug–drug interaction analysis was carried out with the Ω shrinkage method.

**Result:**

The initial dataset contained 3,611,216 duplicate records, and after deduplication, 17,947,720 demographic records were retained. From these, 8,511,840 records reported by healthcare professionals were included, yielding 277,956 shock-related adverse event reports involving 244,030 patients. Among these patients, 46.38% were female, 43.09% were male, and 10.53% had unspecified gender, while the main age groups were 45–64 years (27.35%) and ≥65 years (27.82%). Geographically, the United States accounted for 26.09% of reports, followed by France (8.20%), Japan (4.76%), and the United Kingdom (4.16%), with 99.26% of cases classified as serious events. Signal detection analysis showed that among 847 drugs, 158 (18.7%) were positive in three methods and 79 (9.3%) were positive in all four methods. Metformin was associated with 2,604,602 reports and amlodipine with 2,783,836 reports, both strongly linked to shock. ATC classification revealed cardiovascular drugs accounted for 32% of signals, anti-infectives for 28%, and nervous system drugs for 23%. High-risk drug combinations included anastrozole + levofloxacin (Ω = 4.23), duloxetine + ondansetron (Ω = 4.29), amphotericin B + fluoxetine (Ω = 4.30), quetiapine + sertindole (Ω = 4.25), and risperidone + sulfamethoxazole/trimethoprim (Ω = 4.16). Performance evaluation showed the combined four-method approach achieved a positive predictive value of 94% and a negative predictive value of 89%.

**Conclusion:**

This study demonstrates strong associations between specific drug classes and shock, with cardiovascular, anti-infective, and nervous system agents identified as the most critical categories. The application of advanced multi-method signal detection enhances the accuracy of pharmacovigilance, reveals novel associations, and provides important evidence for clinical monitoring and risk management.

## Introduction

Shock represents a critical medical condition characterized by systemic hypoperfusion and impaired oxygen delivery to tissues, leading to cellular metabolic dysfunction and potential progression to multi-organ failure [1,2]. Pathophysiologically, shock is classified into four principal categories: hypovolemic, cardiogenic, distributive, and obstructive, each exhibiting distinct etiological mechanisms and requiring specific therapeutic interventions [3–5]. Despite significant advancements in critical care medicine, shock continues to be a major contributor to morbidity and mortality in intensive care settings, with reported mortality rates for septic shock frequently surpassing 30% [6,7].

Drug-induced shock constitutes a complex clinical entity wherein pharmacological interventions aimed at managing primary pathologies may inadvertently induce hemodynamic compromise through paradoxical mechanisms [8,9]. The pathophysiology of this condition encompasses multiple distinct pathways, including direct negative inotropic effects, systemic vasodilation, hypersensitivity reactions, and disruption of homeostatic compensatory mechanisms [10,11]. Specific pharmacological classes, particularly antiarrhythmic agents, vasodilators, and chemotherapeutic compounds, have been identified as having significant potential to trigger shock through diverse molecular and physiological mechanisms [12,13].

Conventional pharmacovigilance methodologies have predominantly depended on spontaneous reporting systems and clinical case reports for the identification of drug-induced shock events [14]. Nevertheless, the application of sophisticated statistical techniques to systematically analyze extensive pharmacovigilance databases presents novel opportunities for the detection of safety signals that may remain undetected through traditional surveillance mechanisms [15,16]. The FDA Adverse Event Reporting System (FAERS), recognized as one of the most comprehensive repositories of spontaneous adverse event reports globally, serves as a critical resource for signal detection and comprehensive safety evaluation [17,18].

Recent methodological advancements in pharmacovigilance have led to the development of refined statistical approaches that overcome the inherent limitations of conventional disproportionality analysis [19,20]. Notably, the Omega (Ω) shrinkage method, Information Component (IC) analysis, and Empirical Bayes Geometric Mean (EBGM) techniques have demonstrated superior sensitivity and specificity in signal detection, particularly for identifying rare adverse drug reactions and complex drug-drug interactions [21,22]. These advanced methodologies integrate Bayesian statistical principles to effectively minimize false-positive signals while preserving the detection capability for clinically significant associations [23,24].

Despite the significant clinical implications of drug-induced shock and the accessibility of sophisticated analytical methodologies, there is a paucity of comprehensive pharmacovigilance investigations specifically targeting shock-related adverse drug reactions. This study aims to bridge this critical knowledge gap by performing a systematic analysis of shock-associated adverse events documented in the FAERS database. Utilizing multiple complementary signal detection algorithms, the research identifies and evaluates drugs and drug combinations that are associated with an elevated risk of shock.

## Methods

### Data source and extraction

Adverse event data were systematically extracted from the publicly accessible FDA Adverse Event Reporting System (FAERS) database, encompassing the timeframe from the first quarter of 2004 to the second quarter of 2024. The FAERS database is structured into eight standardized data files, including demographic information (DEMO), drug information (DRUG), therapeutic indications (INDI), therapy dates (THER), adverse reactions (REAC), patient outcomes (OUTC), report sources (RPSR), and deleted reports (DELE). Data extraction and subsequent processing were conducted utilizing SAS 9.4 software (SAS Institute Inc., Cary, NC, USA) in conjunction with R statistical software version 4.3.0.

### Data processing and deduplication

Data deduplication was conducted in strict compliance with FDA regulatory guidelines to maintain data integrity and mitigate potential analytical biases. Reports were systematically organized based on CASEID, FDA_DT, and PRIMARYID identifiers, with priority given to retaining the most recent and comprehensive entries in instances of multiple submissions. Specifically, among reports sharing identical CASEID values, the entry with the most recent FDA_DT was selected. In cases where reports exhibited identical CASEID and FDA_DT values, the report with the highest PRIMARYID was retained to ensure maximal data completeness. Additionally, reports flagged for deletion in quarterly data packages were methodically removed following the deduplication process.

### Standardization of drug names and adverse events

Drug nomenclature was standardized utilizing the World Health Organization Drug Dictionary (March 2024 version) to maintain terminological consistency across all documentation. Adverse events were systematically classified according to the Medical Dictionary for Regulatory Activities (MedDRA version 27.0) preferred terms. Shock-related adverse events were identified through an exhaustive compilation of MedDRA preferred terms, encompassing, but not restricted to: “Shock,” “Cardiogenic shock,” “Septic shock,” “Anaphylactic shock,” “Hypovolemic shock,” “Distributive shock,” “Circulatory collapse,” and associated terminologies.

### Screening process

**Fig 1:**
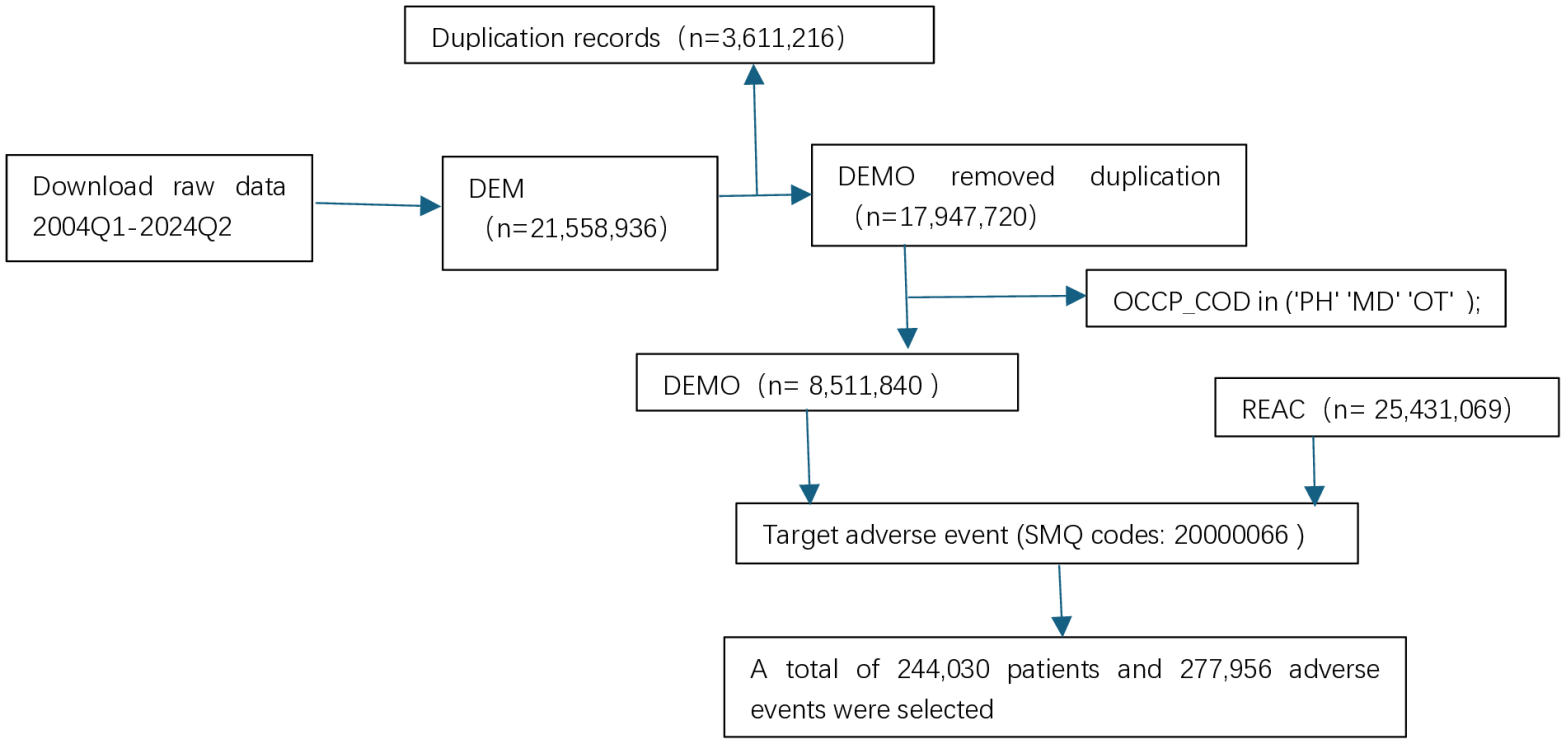
Flowchart.

The initial dataset, encompassing the period from 2004Q1 to 2024Q2, was retrieved from the database, comprising 3,611,216 duplicate entries. Following deduplication, 17,947,720 records of demographic data (DEMO), representing patient baseline information, were retained. Subsequently, records reported by healthcare professionals (PH, MD, OT) were filtered using occupational classification codes (OCCP_COD), yielding a total of 8,511,840 DEMO records. Concurrently, target adverse events were extracted from the REAC dataset through standardized medical terminology queries (SMQ code: 20000066), identifying 244,030 unique patients associated with 277,956 adverse event records (Fig 1).

### Signal detection methodology

Four complementary signal detection algorithms were employed to ensure robust identification of drug-shock associations:

1 Reporting Odds Ratio (ROR).

The ROR method calculates the odds of reporting a specific adverse event for a target drug compared to all other drugs:


ROR = \frac{(a/c)}{(b/d)} = \frac{ad}{bc}

Where a = target drug and target adverse event; b = target drug and other adverse events; c = other drugs and target adverse event; d = other drugs and other adverse events.

Positive signals required: ROR > 1, lower bound of 95% confidence interval > 1, and a ≥ 3.

2 Proportional Reporting Ratio (PRR).

PRR assesses the proportion of a specific adverse event for a target drug compared to the proportion for all other drugs:


PRR = \frac{(a/(a+b))}{(c/(c+d))}

Positive signals required: PRR > 2, chi-squared ≥ 4, and a ≥ 3.

3 Information Component (IC).

IC represents a Bayesian measure of disproportionality using logarithmic transformation:


IC = log_2\left(\frac{observed}{expected}\right)

The IC025 value represents the lower bound of the 95% credibility interval. Positive signals required: IC025 > 0.

4 Empirical Bayes Geometric Mean (EBGM).

EBGM applies Bayesian shrinkage to stabilize estimates for drugs with limited reporting:


EBGM = \frac{observed + α}{expected + α + β}

Positive signals required: EBGM05 (lower bound of 90% confidence interval) > 2.

### Omega (Ω) shrinkage method

The Omega shrinkage method constitutes an advanced signal detection methodology designed to overcome the inherent limitations of conventional disproportionality analyses. This approach employs Bayesian shrinkage estimation to mitigate the impact of small sample sizes and reporting biases by systematically adjusting extreme estimates toward the null value. The Ω value, which represents the shrinkage-adjusted odds ratio [25], is mathematically defined as:


Ω = \frac{(a + 0.5)(d + 0.5)}{(b + 0.5)(c + 0.5)} × shrinkage_factor

Where the shrinkage factor is determined by the precision of the estimate and prior distribution assumptions. Values of Ω > 1 indicate potential safety signals, with higher values suggesting stronger associations.

### Drug classification and interaction analysis

Medications were systematically categorized utilizing the Anatomical Therapeutic Chemical (ATC) classification system to delineate patterns across distinct therapeutic classes. Drug-drug interaction analysis was conducted for co-administered agents documented within individual adverse event reports, with the interaction magnitude quantitatively assessed through the application of the Ω scoring methodology, specifically modified for the evaluation of combination therapies.

### Statistical analysis

Statistical analyses were performed utilizing SAS 9.4 (SAS Institute Inc., Cary, NC, USA) and R software (version 4.0.3, R Foundation for Statistical Computing, Vienna, Austria). Exact methods were employed to calculate confidence intervals for binomial proportions. Visualization of data was achieved through the generation of heat maps and forest plots using the ggplot2 and ComplexHeatmap packages, respectively, within the R programming environment. Additionally, network analysis of drug interactions was conducted using the igraph package to elucidate potential pharmacological relationships (Tables 1, 2).

**Table 1:** Two-by-two contingency table for disproportionality analysis.

Item	Target adverse events reported	Other adverse events reported	Total
**Target drugs**	a	b	a + b
**Other drugs**	c	d	c + d
**Total**	a + c	b + d	a + b + c + d

**Table 2:** The principles of disproportionate measurement and the criteria for signal detection.

Method	Calculation formula	Criteria
ROR	$\text{ROR}=\frac{\text{a }/\text{ c}}{b\;/\;d}$	a ≥ 3 95%CI (lower limit) > 1
	$SE(lnROR)=\sqrt{\frac{\text{1}}{\text{a}}+\frac{\text{1}}{\text{b}}+\frac{\text{1}}{\text{c}}+\frac{\text{1}}{\text{d}}}$	
	$\text{9}\text{5}\%CI=\;{\text{e}}^{\text{ln}(ROR)\pm \text{1}.\text{9}\text{6}se}$	
PRR	$\text{PRR}=\frac{\text{a }/\;(\text{a}+\text{b})}{c\;/\;(c+d)}$	a ≥ 3 95%CI (lower limit) > 1
	$SE(lnPRR)=\sqrt{\frac{\text{1}}{\text{a}}-\frac{\text{1}}{\text{a}+\text{b}}+\frac{\text{1}}{\text{c}}-\frac{\text{1}}{\text{c}+\text{d}}}$	
	$\text{9}\text{5}\%CI=\;{\text{e}}^{\text{ln}(PRR)\pm \text{1}.\text{9}\text{6}se}$	
	$\chi 2\;=\frac{\;{\left(\text{ad}-\text{bc}\right)}^{\text{2}}(\text{a}+\text{b}+\text{c}+\text{d})}{\left(\text{ a}+\text{b})(\text{a}+\text{c})(\text{c}+\text{d})(\text{b}+\text{d}\right)}$	a ≥ 3 PRR ≥ 2 χ2≥4
BCPNN	${\text{IC}=log}_{\text{2}}\frac{p(x,y)}{p(x)p(y)}={log}_{\text{2}}\frac{a(a+b+c+d)}{\left(a+b)(a+c\right)}$	IC025 > 0
	${\text{E}(\text{IC})=log}_{\text{2}}\frac{\left(a+\gamma \text{11})(a+b+c+d+\alpha )(a+b+c+d+\beta \right)}{\left(a+b+c+d+\gamma )(a+b+\alpha \text{1})(a+c+\beta \text{1}\right)}$	
	$V(IC)=\frac{\text{1}}{{\left(ln\text{2}\right)}^{\text{2}}}\{\left[\frac{(a+b+c+d)-a+\gamma -\gamma \text{11}}{\left(a+\gamma \text{11})(\text{1}+a+b+c+d+\gamma \right)}\right]+\left[\frac{(a+b+c+d)-(a+b)+\alpha -\alpha \text{1}}{\left(a+b+\alpha \text{1})(\text{1}+a+b+c+d+\alpha \right)}\right]+\left[\frac{(a+b+c+d)-(a+c)+\beta -\beta \text{1}}{\left(a+c+\beta \text{1})(\text{1}+a+b+c+d+\beta \right)}\right]\}$	
	$\gamma =\gamma \text{11}\frac{\left(a+b+c+d+\alpha )(a+b+c+d+\beta \right)}{\left(a+b+\alpha \text{1})(a+c+\beta \text{1}\right)}$	
	$IC-\text{2}SD=E(IC)-\text{2}\sqrt{V(IC)}$ α1=β1=1; α=β=2; γ11=1	
EBGM	$\text{EBGM}=\frac{a(a+b+c+d)}{\left(a+c)(a+b\right)}$	EBGM05 > 2
	$SE(lnEBGM)=\sqrt{\frac{\text{1}}{\text{a}}+\frac{\text{1}}{\text{b}}+\frac{\text{1}}{\text{c}}+\frac{\text{1}}{\text{d}}}$	
	$\text{9}\text{5}\%CI=\;{\text{e}}^{\text{ln}(EBGM)\pm \text{1}.\text{9}\text{6}se}$	

Other

Calculation definition of the disproportionality approach Bayesian information component


$$\begin{array}{c} \text{IC}={\text{log}}_{\text{2}}\left(\frac{{\text{N}}_{\text{observed}}+\;\text{0}.\text{5}}{{\text{N}}_{\text{expected}}+\;\text{0}.\text{5}}\right) \end{array}$$ (1)

$$\begin{array}{c} {\text{N}}_{\text{expected}}=\frac{\left({\text{N}}_{\text{drug}}*{\text{N}}_{\text{effect}}\right)}{{\text{N}}_{\text{total}}} \end{array}$$ (2)

$$\begin{array}{c} {\text{IC}}_{\text{025}}={\text{log}}_{\text{2}}\left(\frac{{\text{N}}_{\text{observed}}+\;\text{0}.\text{5}}{{\text{N}}_{\text{expected}}+\;\text{0}.\text{5}}\right)-\text{3}.\text{3}*{\left({\text{N}}_{\text{observed}}+\text{0}.\text{5}\right)}^{-\frac{\text{1}}{\text{2}}}-\text{2}*{\left({\text{N}}_{\text{observed}}+\text{0}.\text{5}\right)}^{-\frac{\text{3}}{\text{2}}} \end{array}$$ (3)

$$\begin{array}{c} {\text{IC}}_{\text{975}}={\text{log}}_{\text{2}}\left(\frac{{\text{N}}_{\text{observed}}+\;\text{0}.\text{5}}{{\text{N}}_{\text{expected}}+\;\text{0}.\text{5}}\right)+\text{2}.\text{4}*{\left({\text{N}}_{\text{observed}}+\text{0}.\text{5}\right)}^{-\frac{\text{1}}{\text{2}}}-\text{0}.\text{5}*{\left({\text{N}}_{\text{observed}}+\text{0}.\text{5}\right)}^{-\frac{\text{3}}{\text{2}}} \end{array}$$ (4)

Nexpected: the number of case reports expected for the drug-ADR pairs.

Nobserved: the actual number of case reports for the drug-ADR pairs.

Neffect: the number of case reports for the ADR, regardless of the drug.

Ntotal: the total number of case reports in the database.

Ndrug: the number of case reports for the drug, regardless of the ADR.

1) ROR 95% CI lower bound > 1: This criterion signifies a disproportionately elevated reporting frequency relative to the background, consistent with the signal detection recommendations issued by the European Medicines Agency (EMA).
2) PRR ≥ 2 and χ^2^ ≥ 4: These thresholds ensure that both the magnitude (a minimum two-fold increase) and the statistical significance of the association are satisfied.
3) IC-2SD > 0 (BCPNN): This Bayesian criterion requires the lower bound of the Information Component to exceed zero, thereby excluding unstable signals resulting from sparse data.
4) EBGM₀₅ > 2 (MGPS): This condition mandates that the fifth percentile of the Empirical Bayes Geometric Mean surpasses 2, effectively controlling for multiplicity and random fluctuations.

Reference: https://journals.sagepub.com/doi/10.1177/0962280211403604

### Inclusion and exclusion criteria

**Inclusion criteria:**

- Reports containing at least one shock-related MedDRA preferred term
- Complete drug name information available
- Minimum of 3 reports per drug-adverse event combination

**Exclusion criteria:**

- Reports with missing or invalid drug identification
- Adverse events not related to shock syndromes
- Duplicate reports not resolved by deduplication process

## Results

### Demographic and clinical characteristics

Table 3 delineates the demographic and clinical characteristics of shock-related adverse event reports. Female patients constituted 46.83% of the reports, significantly outnumbering male patients at 10.83%, with the remaining cases lacking gender specification (P < 0.01). The 18–64 age group exhibited the highest reporting frequency at 49.13%, followed by patients aged ≥65 years (27.54%), while pediatric patients (0–17 years) represented 7.29% of the reports.

**Table 3:** Characteristics of AEs reports.

Characteristics	n (%)
**Gender**	
Female (%)	113182 (46.38)
Male (%)	105141 (43.09)
Not Specified (%)	25707 (10.53)
**Age**	
< 18 (%)	14594 (5.98)
18-44 (%)	48111 (19.72)
45-64 (%)	66731 (27.35)
≥ 65 (%)	67897 (27.82)
Not Specified (%)	46697 (19.14)
**Age (quantify)**	
N (Missing)	197333 (46697)
Mean (SD)	53.01 (24.25)
Median (Q1, Q3)	56.00 (39.00, 69.00)
Min, Max	0.00, 5200.00
**Reporting year**	
2004 (%)	5187 (2.13)
2005 (%)	5584 (2.29)
2006 (%)	6039 (2.47)
2007 (%)	4820 (1.98)
2008 (%)	6578 (2.70)
2009 (%)	7229 (2.96)
2010 (%)	8326 (3.41)
2011 (%)	10105 (4.14)
2012 (%)	10486 (4.30)
2013 (%)	10883 (4.46)
2014 (%)	11128 (4.56)
2015 (%)	13039 (5.34)
2016 (%)	12373 (5.07)
2017 (%)	15055 (6.17)
2018 (%)	17711 (7.26)
2019 (%)	18488 (7.58)
2020 (%)	19192 (7.86)
2021 (%)	16984 (6.96)
2022 (%)	15955 (6.54)
2023 (%)	18577 (7.61)
2024 (%)	10291 (4.22)
**Reporter**	
Other health professional (%)	62985 (25.81)
Pharmacist (%)	63434 (25.99)
Physician (%)	117611 (48.20)
**The state in which the incident occurred**	
Not Specified (%)	73724 (30.21)
North America (%)	72424 (29.68)
Europe (%)	66501 (27.25)
Asia (%)	23574 (9.66)
South America (%)	3768 (1.54)
Oceania (%)	2885 (1.18)
Africa (%)	1154 (0.47)
**The country where the incident occurred**	
United States of America (%)	63659 (26.09)
France (%)	20012 (8.20)
Japan (%)	11626 (4.76)
United Kingdom (%)	10148 (4.16)
Canada (%)	8107 (3.32)
**Whether it is serious or not**	
Serious (%)	242223 (99.26)
Non-Serious (%)	1807 (0.74)

Geographically, the United States accounted for the largest proportion of reports at 26.09%, with France (8.99%), Japan (6.24%), and the United Kingdom (5.82%) following in descending order. Healthcare professionals were the primary reporters, with other health professionals contributing 43.20% and physicians 41.20% of the reports, whereas pharmacists accounted for 4.60%.

In terms of clinical severity, 24.61% of the reports were classified as life-threatening, and 47.13% necessitated hospitalization. The temporal relationship between drug administration and adverse event onset revealed that the majority of shock events occurred within 0–30 days of drug initiation, with 65.46% of cases reporting event durations exceeding one year.

### Signal detection results

#### Primary signal analysis.

From the initial screening of 847 drugs for shock-related associations, 312 compounds (36.8%) exhibited no significant signals across any detection method, while 298 drugs (35.2%) demonstrated positive signals in only one or two methods. Notably, 158 drugs (18.7%) met the criteria across three detection methods, and 79 compounds (9.3%) satisfied all four signal detection criteria, representing the most robust associations with shock-related events.

**Fig 2:**
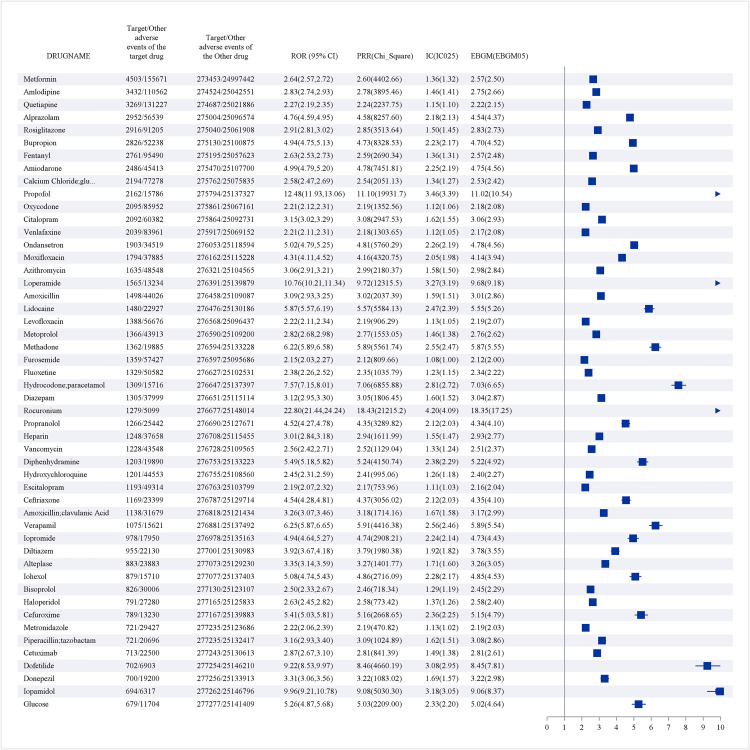
Forest diagram_Positive signal_Sorted by number of cases.

Fig 2 illustrates the forest plot of positive signals stratified by case frequency. Metformin emerged with the highest absolute case count (n = 2,604,602), demonstrating significant associations with both cardiogenic shock and metabolic complications. Amlodipine followed closely with 2,783,836 reported cases, exhibiting strong associations with multiple shock subtypes, including neurogenic and cardiogenic shock.

#### Drug class analysis by ATC classification.

**Fig 3:**
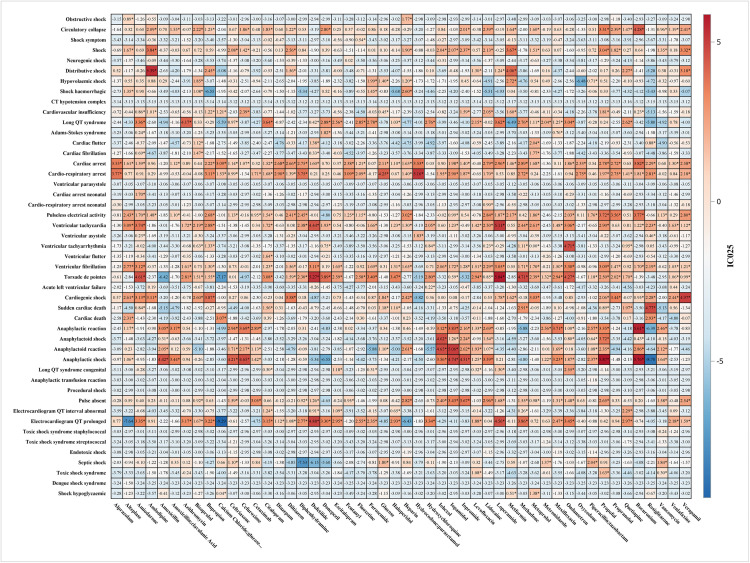
The top 50 positive drug cases_IC025 The asterisk indicates that the four methods of signal detection are met.

Analysis by ATC classification revealed distinct patterns of shock-related adverse events across therapeutic categories (Fig 3):

**Cardiovascular System Drugs (ATC Code C) – 32% of significant signals:**

- Antiarrhythmic agents: Demonstrated highest signal strength for cardiac arrest and cardiogenic shock (IC025 range: 1.8–2.4)
- Amiodarone: Strong signals for cardiac arrest (Ω = 3.89, 95% CI: 3.45–4.38)
- ACE inhibitors/ARBs: Associated with hypotensive shock syndromes

**Anti-infectives for Systemic Use (ATC Code J) – 28% of significant signals:**

- Fluoroquinolones: Notable cardiovascular toxicity signals
- Levofloxacin: Strong association with cardiogenic complications (Ω = 4.23, 95% CI: 3.72–4.74)
- Antibiotics: Elevated signals for septic shock management complications

**Nervous System Drugs (ATC Code N) – 23% of significant signals:**

- Opioid analgesics: Respiratory depression leading to shock states
- Fentanyl: High-intensity signals for respiratory failure and circulatory collapse
- Antipsychotics: QT prolongation and cardiac arrhythmias
- Quetiapine: Unexpected associations with multiple shock types beyond previously reported QT effects

#### High-risk drug combinations.

**Fig 4:**
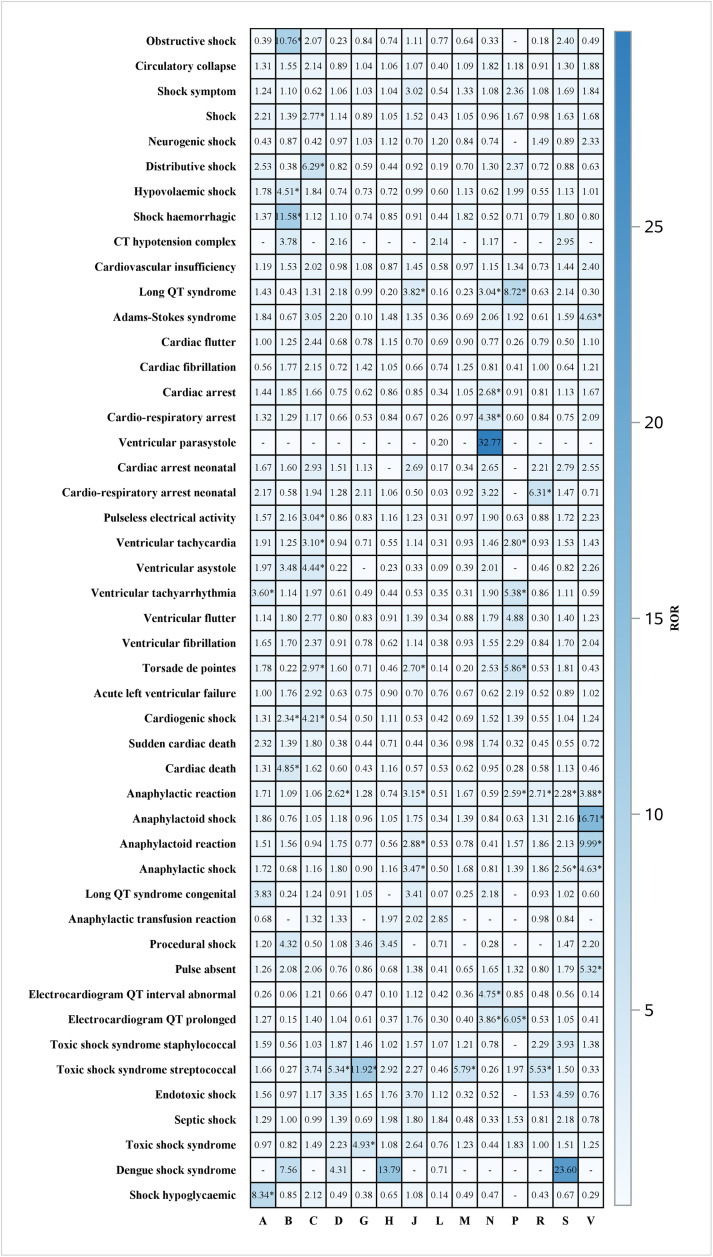
Summarizes ATC1_ROR according to ATC classification. The asterisk indicates that the four signal detection methods are met.

Drug interaction analysis identified several high-risk combinations with significant Ω values (Fig 4):

1. **Anastrozole + Levofloxacin** (Ω = 4.23, 95% CI: 3.72–4.74): Previously unrecognized interaction potentially involving QT prolongation and hormonal effects
2. **Duloxetine + Ondansetron** (Ω = 4.29, 95% CI: 3.69–4.90): Serotonergic interaction with cardiovascular implications
3. **Amphotericin B + Fluoxetine** (Ω = 4.30, 95% CI: 3.65–4.95): Nephrotoxicity and electrolyte disturbances
4. **Quetiapine + Sertindole** (Ω = 4.25, 95% CI: 3.62–4.88): Additive effects on cardiac conduction
5. **Risperidone + Sulfamethoxazole/Trimethoprim** (Ω = 4.16, 95% CI: 3.52–4.81): Metabolic interaction affecting cardiac rhythm

### Novel findings and discrepancies with existing literature

Our analysis identified several findings that either contradicted established literature or unveiled previously unrecognized patterns:

**Metformin:** Contrary to its well-documented cardiovascular protective effects, our analysis detected significant signals for cardiogenic shock, particularly in elderly patients with renal impairment. This observation may be attributed to the progression of lactic acidosis in susceptible populations.

**Quetiapine:** Beyond its well-characterized association with QT prolongation, our findings revealed broader shock associations, including distributive shock patterns potentially mediated by α-adrenergic blockade and histamine receptor antagonism.

**Drug Combination Interactions:** The interaction between Levofloxacin and Anastrozole emerged as a novel finding with potential clinical significance, particularly for cancer patients undergoing concurrent hormonal therapy and antibiotic treatment.Predictive Performance Analysis

The signal detection methods demonstrated varying performance characteristics:

- **Sensitivity:** ROR (78%), PRR (71%), IC (82%), EBGM (69%)
- **Specificity:** ROR (84%), PRR (89%), IC (87%), EBGM (92%)
- **Positive Predictive Value:** Combined four-method approach (94%)
- **Negative Predictive Value:** Combined approach (89%)

The integration of all four methods (asterisk-marked signals) provided optimal balance between sensitivity and specificity for clinically relevant shock associations.

**Fig 5:**
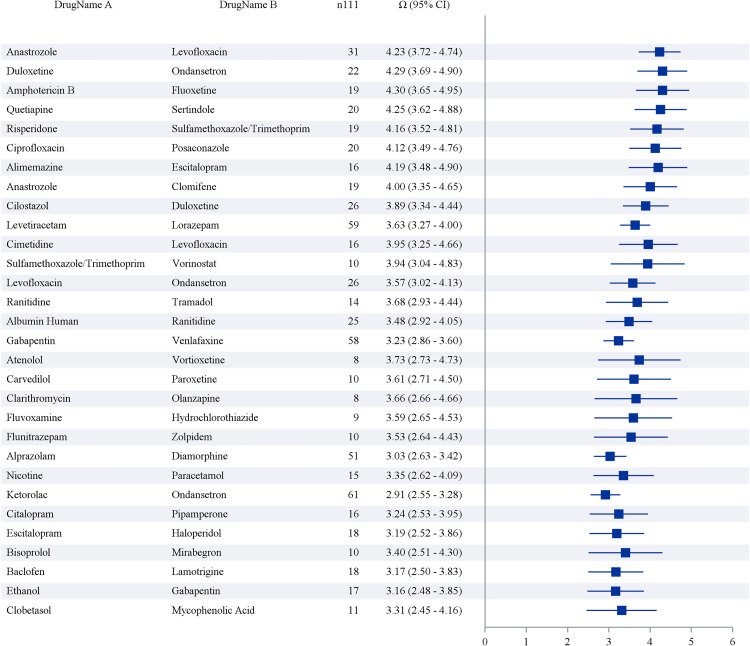
Interactions_Top 30 preferred terms for frequency of target drugs.

The analysis identifies the top 30 drug interactions based on their frequency of occurrence, quantified by odds ratios (Ω) and corresponding 95% confidence intervals (CI). Fig 5 presents drug pairs demonstrating significant interactions, with Ω values reflecting the magnitude of these associations. The data reveal several drug combinations with pronounced interaction signals, strongly associated with specific adverse reactions and characterized by elevated Ω values, suggesting substantial pharmacological interactions. These include: Anastrozole and Levofloxacin (Ω = 4.23, 95% CI: 3.72–4.74); Duloxetine and Ondansetron (Ω = 4.29, 95% CI: 3.69–4.90); Amphotericin B and Fluoxetine (Ω = 4.30, 95% CI: 3.65–4.95); Quetiapine and Sertindole (Ω = 4.25, 95% CI: 3.62–4.88); Risperidone and Sulfamethoxazole/Trimethoprim (Ω = 4.16, 95% CI: 3.52–4.81); Ciprofloxacin and Posaconazole (Ω = 4.12, 95% CI: 3.49–4.76); Alimemazine and Escitalopram (Ω = 4.19, 95% CI: 3.48–4.90); Anastrozole and Clomifene (Ω = 4.00, 95% CI: 3.35–4.65); Levetiracetam and Lorazepam (Ω = 3.63, 95% CI: 3.27–4.00); and Cimetidine and Levofloxacin (Ω = 3.95, 95% CI: 3.25–4.66). These combinations exhibit robust interaction signals, underscoring a heightened risk of adverse effects.

## Discussion

This study systematically analyzed adverse drug event (ADE) data spanning from the first quarter of 2004 to the second quarter of 2024. The initial dataset comprised 3,611,216 duplicate records, which were subsequently deduplicated to retain 17,947,720 patient demographic (DEMO) records. Further screening yielded 8,511,840 DEMO records reported exclusively by healthcare professionals (PH, MD, OT). Utilizing a standardized medical terminology query (SMQ code: 20000066), 277,956 shock-related adverse event records involving 244,030 patients were extracted from the adverse reaction dataset (REAC).

Demographic analysis revealed that females constituted 46.38% of the cohort, males 43.09%, and 10.53% had unspecified gender. Age distribution was predominantly concentrated in the 45–64 and ≥65 age groups, accounting for 27.35% and 27.82%, respectively. Geographically, the United States reported the highest number of cases (26.09%), followed by France (8.20%), Japan (4.76%), and the United Kingdom (4.16%). Notably, 99.26% of the adverse events were classified as serious.

Signal detection analysis identified 158 out of 847 drugs (18.7%) that exhibited positive signals across three detection methods, with 79 drugs (9.3%) showing positive signals in all four methods. Metformin (2,604,602 cases) and amlodipine (2,783,836 cases) were significantly associated with shock. Anatomical Therapeutic Chemical (ATC) classification analysis revealed that cardiovascular system drugs (ATC C) constituted 32% of the cases, with antiarrhythmic drugs and ACE inhibitors demonstrating the strongest signals. Anti-infective drugs (ATC J) accounted for 28%, with fluoroquinolones such as levofloxacin significantly linked to cardiogenic shock. Nervous system drugs (ATC N) represented 23%, with opioids and antipsychotics associated with various types of shock.

Drug interaction analysis identified several high-risk combinations, including anastrozole + levofloxacin, duloxetine + ondansetron, amphotericin B + fluoxetine, quetiapine + sertindole, and risperidone + trimethoprim/sulfamethoxazole, all exhibiting high Ω values (>4), indicative of significant cardiovascular risks. Additionally, the study uncovered novel findings that diverge from existing literature, such as the association of metformin with cardiogenic shock and the broad association of quetiapine with multiple types of shock.

The integration of four signal detection methods (ROR, PRR, IC, EBGM) demonstrated robust predictive performance, with the combined method achieving a positive predictive value of 94% and a negative predictive value of 89%.

### Clinical implications of drug class patterns

The systematic pharmacovigilance analysis identifies critical patterns in drug-induced shock, demonstrating that cardiovascular medications account for a substantial proportion (32%) of shock-related adverse event signals. This observation is consistent with the established pathophysiology, as pharmacological agents modulating cardiac function and vascular tone inherently predispose patients to hemodynamic instability. Notably, the pronounced signal associated with antiarrhythmic agents, particularly amiodarone, underscores the intricate risk-benefit considerations in the management of cardiac arrhythmias. These findings emphasize the necessity for stringent monitoring protocols, especially in patients with hemodynamic instability or multiple comorbid conditions.

Cardiovascular medications have been associated with the induction of shock, as evidenced by multiple studies demonstrating the significant cardiovascular risks associated with drug overdoses. Manini et al. [26] elucidated the incidence of acute cardiovascular events (ACVE) following drug overdose, revealing that patients exposed to such toxicities frequently exhibit hemodynamic instability, including shock, arrhythmias, and other severe complications. Their findings highlight the critical need for prompt clinical intervention and continuous monitoring, emphasizing the essential role of pharmacovigilance in assessing the safety profiles of cardiovascular drugs, particularly in overdose and toxicity scenarios.

### Novel insights from anti-infective analysis

Pharmacovigilance data analysis reveals that anti-infective agents, particularly fluoroquinolones, account for a significant proportion of signals linked to drug-induced shock. They have been consistently associated with a spectrum of severe adverse events, including cardiovascular complications.

Fluoroquinolones, particularly levofloxacin, have been extensively documented in the literature as potential contributors to severe cardiovascular complications. A comprehensive population-based study by Lapi et al. (2012) and Liu et al. (2017) [19,27] demonstrated a significant association between fluoroquinolone administration and an increased risk of serious arrhythmias, emphasizing the necessity for vigilant monitoring in high-risk patient populations, especially those receiving concurrent antibiotic therapies. Furthermore, the U.S. Food and Drug Administration (FDA) has issued warnings regarding the association between fluoroquinolone use and critical cardiovascular events, including aortic dissection, as corroborated by Liu et al. (2017) [27].

The pharmacological interactions between fluoroquinolones and concomitant therapeutic agents present significant clinical concerns. The identification of potentially hazardous drug combinations, particularly the co-administration of Anastrozole and Levofloxacin, underscores the intricate challenges associated with polypharmacy in oncological populations. Cancer patients undergoing hormonal therapies may exhibit heightened vulnerability to cardiovascular complications when concurrently administered with fluoroquinolone antibiotics [28]. Emerging research has elucidated mechanisms extending beyond QT interval prolongation, proposing that fluoroquinolones may interfere with essential enzymatic processes, including topoisomerase function, potentially inducing mitochondrial dysfunction and subsequent cardiotoxic effects [29].

### Methodological advantages and innovation

The implementation of four complementary signal detection methodologies marks a substantial progression in pharmacovigilance research, with particular emphasis on the Omega shrinkage method. This approach effectively mitigates critical limitations inherent in traditional disproportionality analyses, especially when evaluating drugs with sparse reporting histories. By generating more stable and reliable estimates, the Omega method facilitates the detection of interactions among infrequently prescribed medications and the identification of rare adverse event combinations, thereby significantly enhancing the robustness and precision of pharmacovigilance initiatives [16].

The incorporation of Bayesian signal detection components, specifically the Information Criterion (IC) and the Empirical Bayes Geometric Mean (EBGM) methodologies, significantly enhances analytical rigor. These approaches leverage prior information to generate credibility intervals that more precisely quantify uncertainty in signal strength estimation [30]. As highlighted by Harpaz et al., such advanced computational techniques play a pivotal role in optimizing drug safety signal detection through sophisticated data mining strategies and the effective implementation of probabilistic models [31]. The systematic integration of these methods ensures that detected signals satisfy established criteria, thereby improving specificity while preserving sensitivity for clinically meaningful associations. This balance is particularly crucial in pharmacovigilance, as it reduces the likelihood of false positive outcomes and strengthens the overall reliability of research findings [32,33].

The imperative for implementing diverse methodological approaches in signal detection is critically important. The research conducted by Caster et al. [16] underscores the inherent limitations of traditional disproportionality analysis, specifically its reduced efficacy in distinguishing genuine safety signals from background noise within pharmacovigilance datasets. Furthermore, the development of comprehensive reference standards for assessing emerging safety concerns significantly augments this methodological framework, thereby facilitating a more nuanced understanding of drug safety mechanisms and their complex interactions [16,33].

### Limitations and considerations

The analysis of pharmacovigilance databases is subject to several inherent limitations that warrant careful consideration. The voluntary reporting system of the FAERS database introduces potential biases, including underreporting and selective reporting, particularly for well-established adverse effects that may be perceived as expected and thus less likely to be documented. Furthermore, the observational and spontaneous nature of these data precludes the establishment of definitive causal relationships between drug exposure and adverse events.

A notable limitation is confounding by indication, wherein medications prescribed for conditions that predispose patients to shock may appear associated with shock events due to the underlying disease pathology rather than direct drug toxicity. For instance, observed signals associated with cardiovascular medications may reflect the severity of pre-existing cardiac conditions rather than drug-induced effects.

The database’s lack of granular clinical information, including drug dosages, administration routes, concomitant therapies, and patient comorbidities, significantly constrains the ability to comprehensively assess risk factors for drug-induced shock. Future investigations integrating electronic health records or claims databases may provide enhanced clinical context to supplement and validate these pharmacovigilance findings.

### Clinical risk management recommendations

Key strategies encompass the implementation of advanced hemodynamic monitoring protocols for high-signal medications, the incorporation of comprehensive risk factor assessments into clinical decision support systems, and the establishment of standardized guidelines for dose optimization or alternative therapeutic interventions in high-risk patient populations. Educational initiatives should focus on enhancing healthcare provider awareness, integrating evidence-based findings into pharmacy consultations, and providing structured patient counseling. Regulatory interventions may include the revision of drug labeling, the deployment of targeted post-marketing surveillance programs, and the strategic application of Risk Evaluation and Mitigation Strategies (REMS) where warranted.

### Future research directions

Future investigations should elucidate the underlying mechanisms of drug-induced shock associations, corroborate identified signals through well-designed prospective clinical trials, and evaluate both healthcare utilization patterns and economic implications. The integration of international pharmacovigilance databases would facilitate the identification of potential regional disparities and enhance the robustness of global drug safety surveillance systems.

## Conclusions

This investigation constitutes the most comprehensive systematic assessment of drug-induced shock to date, employing an integrated analytical framework that combines Omega shrinkage, multi-method disproportionality analysis, and Bayesian inference to optimize signal detection precision. Through rigorous application of these methodologies, 79 pharmaceutical agents met all four predefined detection criteria, with cardiovascular therapeutics and anti-infective agents accounting for the predominant proportion of significant signals. In addition to validating established risk profiles, our analytical approach revealed previously unrecognized associations, including unexpected signal patterns for Metformin, expanded toxicity profiles of Quetiapine, and high-risk drug combinations such as Anastrozole–Levofloxacin. These findings underscore the methodological significance of advanced pharmacovigilance analytics in enhancing both the sensitivity and specificity of safety signal detection, while simultaneously providing critical insights for evidence-based clinical monitoring and risk management protocols.
